# Assessing the Clinical Requirement of 2.5% Phenylephrine for Diagnostic Pupil Examination

**DOI:** 10.1089/jop.2020.0111

**Published:** 2021-06-03

**Authors:** Junsang Cho, Brent Bruck, James C. Liu, Susan M. Culican

**Affiliations:** ^1^ University of Missouri, School of Medicine, Columbia, Missouri, USA.; ^2^ Department of Ophthalmology and Visual Sciences, Washington University School of Medicine, St. Louis, Missouri, USA.; ^3^ Department of Ophthalmology, Duke University, Raleigh, North Carolina, USA.; ^4^ Department of Ophthalmology and Visual Neurosciences, University of Minnesota Medical School, Minneapolis, Minnesota, USA.

**Keywords:** dilating agent, eye examination, clinical utility, phenylephrine, tropicamide

## Abstract

***Purpose:*** To evaluate whether the standard dilating drop regimen consisting of phenylephrine, tropicamide, and proparacaine produces clinically significant improvement in pupil size compared to tropicamide and proparacaine during diagnostic eye examination.

***Methods:*** Sixty-three adult patients at Washington University School of Medicine Eye Clinic were enrolled in this prospective, randomized trial. Each patient received one of two dilating drop regimens: phenylephrine + tropicamide + proparacaine (PE+T+PP), which is considered the standard therapy, or tropicamide + proparacaine (T+PP). Main outcome measures were the proportion of pupils able to achieve successful clinical examination without need for additional dilating drops and change in predilation to postdilation pupil size. Comparisons were made using McNemar's test, repeated measures analysis of variance, and Fisher's test to determine whether PE is a necessary component of the standard eye examination.

***Results:*** There were no statistically significant differences between the PE+T+PP and T+PE treatment groups in predilation to postdilation changes in average resting pupil size (1.58 ± 0.66 and 2.61 ± 0.79; *P* = 0.57) or constricted pupil size (2.52 ± 0.93 and 3.56 ± 0.96; *P* = 0.15). There was no statistically significant difference between patients who obtained a successful dilated pupil examination between those receiving PE+T+PP and those receiving T+PP as determined by the examining physicians (Fisher's, *P* = 0.67).

***Conclusion:*** The addition of phenylephrine to tropicamide and proparacaine did not improve pupillary dilation size or ability to conduct a clinical examination. A single dilating agent using tropicamide should be considered in clinical practice.

## Introduction

Pupil dilation is essential for examination of important ophthalmic structures, including the optic nerve and retina. It is estimated that there are currently about 100 million people with diabetic retinopathy, 61 million with glaucoma (open and closed angle combined), and 170 million with age-related macular degeneration worldwide, who are recommended to have regular dilated fundus examinations.^1,2^

Mydriasis is dependent on the action of the pupillary sphincter and dilatory muscles, which are controlled by parasympathetic nerves and sympathetic nerves, respectively. The parasympathetic antagonist tropicamide and sympathetic agonist phenylephrine have commonly been used as a dual-drop regimen to achieve the adequate pupillary dilation necessary for ophthalmic evaluation. The clinical utility of this dual-drop regimen has become increasingly relevant in light of a rise in phenylephrine prices. The cost of phenylephrine has fluctuated over the past decade, with its peak cost at $140 per 15 mL bottle.^3^ This was, in part, due to Food and Drug Administration (FDA) approval of phenylephrine as a “new” drug, marking an end to the drug's generic status and forcing several manufactures without FDA approval to withdraw from the market. The increase in prices has forced many institutions to reconsider the cost-effectiveness and clinical necessity of phenylephrine. Unfortunately, there is scarcity of published data regarding the efficacy of tropicamide alone compared to tropicamide and phenylephrine in a clinic population.

Liu et al.^4^ compared three eye drop regimens: 1% tropicamide +2.5% phenylephrine +0.5% proparacaine, 1% tropicamide +0.5% proparacaine, and 1% tropicamide alone in healthy controls. The study found that the addition of phenylephrine produced a statistically larger difference in dilation of 0.3 mm, but no statistical difference was observed in each regimen's ability to achieve adequate pupil dilation of >7 mm. The authors noted that the study was intended to introduce the idea of a single dilating agent as a possible standard practice in normal situations instead of a dual-drop regimen. In addition, the study ascertained that it may be significantly cheaper to use tropicamide and proparacaine for the dilated examination, while reserving phenylephrine for surgical procedures and for less reactive pupils.^4^

While the statistical benefit of using phenylephrine was demonstrated in the aforementioned pilot study, it did not address whether physicians themselves noted any difference in the use of phenylephrine plus tropicamide versus tropicamide alone in the clinic setting.^5–7^ We hypothesize that the use of a single-drop regimen of tropicamide without phenylephrine is sufficient to provide complete ophthalmic evaluation in the clinical setting.

## Methods

This prospective, randomized study was conducted at the Washington University School of Medicine (WUSM). The study was compliant with the Health Insurance Portability and Accountability Act and approved by the Institutional Review Board (IRB). The research performed followed the tenets of the Declaration of Helsinki and informed consent was obtained from all participants. Participants were recruited during routine ophthalmology visits at WUSM eye clinics.

Exclusion criteria included age <18 years old, any pupillary abnormality that prohibits normal pupillary dilation (surgical pupil, nonreactive pupil, etc.), or patients taking medications known to affect pupil size ascertained by the technician. The inclusion criteria required patients to have a planned dilated examination during their clinical visit. Patients who met these criteria were recruited for the study and consented by a study team member.

**FIG. 1.**
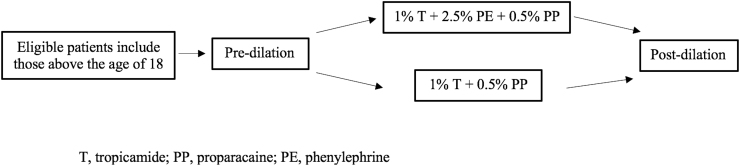
Sequence of dilating regimen administration.

The study team member obtained automated pupillary measurements with a NeurOptics PLR-200 pupillometer (Neuroptics, Laguna Hills, CA), which utilized a self-contained infrared illumination source and internal digital camera to record pupil size. After measuring baseline resting pupil diameter, a standardized light stimulus (180 μW and duration of 30 ms) was presented to stimulate pupil constriction. Constricted pupil diameter was then measured at the point of maximal pupil constriction. Randomization into the respective treatment groups was determined by a coin flip. Eye drop regimens consisted of either 2.5% phenylephrine (PE) hydrochloride +1% tropicamide (T) +0.5% proparacaine (PP) hydrochloride or 1% T + 0.5% PP (Fig. 1).

To minimize disruption to clinical workflows, drops were administered by technicians following their standard clinical practice in the patient office as follows: PP was always administered as the first drop to achieve anesthesia before instilling the cycloplegic. An interval of ∼15 s was used between each eye drop. In the dual-dilating drop regimen, the technicians administered T and PE in arbitrary order consistent with their standard clinical practice.^8,9^ Resting and constricted pupil sizes were again measured between 30 and 60 min after pupillary dilation (postdilation pupil size).

After postdilation pupil measurements were taken, patients proceeded to their normal clinic visit and clinicians indicated whether dilation was adequate for complete dilated fundus examination. Treating clinicians were masked to dilation regimen. If the dilation was deemed inadequate, dilation was deemed a failure and the participant was redilated with the standard protocol (PE+T+PP). Subsequent examination by the clinician was made at a time interval determined at the discretion of the examining physician.

Primary outcome measures included predilation and postdilation resting and constricted pupil size, proportion of pupils able to achieve a postdilation constricted pupil size >7 mm, and clinical efficacy of dilation. Clinical efficacy was determined by the examining physician (masked to dilating regimen) and whether he or she was able to perform an adequate pupil examination of both eyes without requesting additional dilating drops.

### Statistical analysis

Demographic factors were compared using Welch's or Mann–Whitney *U*-test, Chi-square, and Fisher's test. Repeated-measures analysis of variance was used to determine significance of the change in predilation to postdilation pupil size. The dependent variable is time (predilation to postdilation) with eye (right eye [OD]; and left eye [OS]) and pupil state (resting or constricted) represented as within-subject covariates. McNemar's test was used to compare proportion of eyes able to achieve constricted pupil size >7 mm. Although both 6 and 7 mm have been used in the literature as values considered to represent adequate pupillary dilation for diagnostic and surgical settings, 7 mm was used as a more conservative measure and similar to our analysis in the pilot study.^10–15^ Fisher's test was used to determine the clinical benefit of PE+PP+T versus PP + T by evaluation of a successful clinical examination, in addition to relationship of demographic factors and success rates.

Data were analyzed using SAS version 9.3. All data were completely de-identified; participants were identified by a numerical assignment in accordance with the IRB protocol.

## Results

A total of 63 participants were included in this study with an age range of 23–82 years and a mean of 60 ± 12 years. Baseline characteristics were comparable in both groups, including age, gender, race, eye color, and predilation pupil measurements (*P* > 0.05). The majority of patients in both groups were either Caucasian or African American with respect to race and the predominant eye color was brown. The predilation resting and constricted pupil size for OD and OS were comparable. Demographic information and predilation measurements are presented in Table 1.

**Table 1.** Demographic Information and Predilation Measurements

	PE+T+PP	T+PP	*P*
No. of participants	32	31	
Age, years (mean ± SD)	58.12 ± 14.0	61.27 ± 10.11	0.32
Sex (M:F)	12:20	10:21	0.79
Race, *n* (%)			0.57
Asian	1 (3)	0 (0)	
White	8 (25)	8 (26)	
Black	23 (72)	22 (71)	
Latino	0 (0)	1 (3)	
Eye color, *n* (%)			0.78
Brown	23 (72)	23 (74)	
Blue	7 (22)	6 (19)	
Hazel	2 (6)	2 (7)	
Predilation measurements, mm, mean ± SD (range)			
Resting pupil size OD	4.43 ± 1.17 (2.4–6.5)	4.11 ± 0.96 (1.7–6.1)	0.25
Resting pupil size OS	4.48 ± 1.16 (2.6–6.3)	4.14 ± 1.00 (1.6–6.3)	0.22
Constricted pupil size OD	3.14 ± 1.05 (1.3–5.1)	2.97 ± 0.86 (1.3–4.8)	0.50
Constricted pupil size OS	3.01 ± 1.01 (0.5–5.1)	3.02 ± 0.77 (1.2–4.9)	0.99

^*^ Significance was determined using Welch's or Mann–Whitney *U*-test, Chi-square, and Fisher's test.

F, female; M, male; OD, right eye; OS, left eye; PE, phenylephrine; PP, proparacaine; SD, standard deviation; T, tropicamide.

Our primary outcome was analyzing differences in clinical efficacy of the two regimens. With regard to clinical efficacy, there was no statistically significant difference between PE+T+PP and T+PP in achieving successful dilated pupil examinations determined by the residing physician (Fisher's, *P* = 0.67). Among patients receiving PE+T+PP, 30 of the 32 patients (94%) obtained a successful dilated pupil examination as determined by the physician. Patients receiving T+PP had similar success as 28 of the 31 patients (90%) received a successful clinical examination of the fundus (Table 2). The three patients receiving T+PP and two patients receiving PE+T+PP, who initially had unsuccessful clinical examination, eventually underwent re-dilation with standard protocol (triple cocktail) and obtained adequate dilation.

**Table 2.** Proportion of Pupils with Successful Dilated Clinical Examination for PE+T+PP and T+PP

	PE+T+PP	T+PP	*P*
Successful clinical exam, *n* (%)	30 (93.8)	28 (90.3)	0.67
Unsuccessful clinical exam, *n* (%)	2 (6.2)	3 (9.7)	

^*^ Significance was determined using Fisher's test and *P* value <0.05.

Demographic factors, including gender (*P* = 0.92), eye color (*P* = 0.19), age (*P* = 0.61), and race (*P* = 0.35), did not affect clinical efficacy. Because the study was conducted during normal clinic visits, we also measured the time between drop administration and second pupillometry measurement, which corresponded with the time of physician assessment. The mean duration between time of dilation and time of pupil examination was comparable between patients receiving PE+T+PP (51.27 ± 21.94 min) and T+PP (52.72 ± 15.93 min) (*P* > 0.05).

Secondary outcomes included absolute changes in pupil size and the proportion of eyes able to achieve constricted pupil size >7 mm. There was no statistically significant difference in predilation pupil size for both eyes between treatment groups (*P* > 0.05). Despite larger postdilation pupil size in those receiving T+PP than PE+T+PP (average resting 6.73 ± 0.98 vs. 6.06 ± 1.16 and average constricted 6.56 ± 1.09 vs. 5.65 ± 1.44, respectively) and a larger difference in predilation to postdilation pupil size in the T+PP cohort, this difference was not significant (Table 3).

**Table 3.** Postdilation Net Change in Pupil Size of OD and OS

	Resting pupil OD	Resting pupil OS	Resting average (OD+OS)	Constricted pupil OD	Constricted pupil OS	Constricted average (OD+OS)
Predilation to postdilation change in pupil size in mm, mean ± SD (range)						
PE+T+PP	1.59 ± 0.85 (0.5–3.1)	1.62 ± 0.75 (0.4–2.9)	1.58 ± 0.66 (0.3–2.7)	2.41 ± 1.02 (0.5–3.9)	2.61 ± 1.04 (0.8–4.5)	2.52 ± 0.93 (0.4–3.9)
T+PP	2.68 ± 0.90 (0.4–4.2)	2.57 ± 0.88 (0.5–4.4)	2.61 ± 0.79 (1.1–4.1)	3.62 ± 1.25 (0.5–5.8)	3.59 ± 0.96 (0.5–5.4)	3.56 ± 0.96 (1.5–5.1)
P^*^	0.46	0.70	0.57	0.12	0.16	0.15

^*^ Significance determined using repeated-measures analysis of variance test and *P* value <0.05.

The proportion of subjects achieving a threshold of 7 mm postdilation constricted pupil size differed in OD and OS between groups driven mostly by a smaller number of OD eyes achieving >7 mm dilation in the PE+T+PP group (Table 4). In OD, 5 out of 32 (16%) patients receiving PE+T+PP achieved >7 mm dilation, while 14 out of 31 receiving T+PP (45%) achieved the same threshold (McNemar's, *P* < 0.05).

**Table 4.** Proportion of Constricted Pupils That Dilated >7 mm for PE+T+PP and T+PP

	PE+T+PP	T+PP	*P*
OD, *n* (%)			0.04^*^
≤7 mm	27 (84.4)	17 (54.8)	
>7 mm	5 (15.6)	14 (45.2)	
OS, *n* (%)			0.172
≤7 mm	21 (65.6)	18 (58.0)	
>7 mm	11 (34.4)	13 (41.9)	

^*^ Significance was determined using McNemar's test and a *P* value <0.05.

## Discussion

Over the past decade, phenylephrine prices have increased as much as 50-fold at WUSM Eye Center and other centers across the country. With prices prone to fluctuations, the use of phenylephrine for routine diagnostic examination should be considered carefully. Liu et al.^4^ reported that despite a statistically significant difference in mean pupillary dilation between PE+T+PP versus T+PP in a group of healthy volunteers, the mean difference in the postdilation pupil size was only 0.3 mm between the groups. In addition, there was no significant difference in the proportion of pupils able to achieve a target of >7 mm. These findings point toward a negligible benefit in the use of phenylephrine during routine dilation examinations.

The use of phenylephrine increases the cost to conduct the examination and exposes patients to potential unwanted side effects, including systemic cardiovascular effects, allergic reactions, and acute angle closure glaucoma.^16–20^ Ramsali et al.^20^ reported a patient who sustained a subarachnoid hemorrhage from systemic hypertension induced by phenylephrine use and noted that topical phenylephrine should be used cautiously, especially in elderly patients, with appropriate monitoring of hemodynamic status.^21^

This study sought to address some of the shortcomings of the previous study regarding the efficacy of PE+T+PP compared to T+PP by using a larger, clinical patient population rather than a cohort of healthy volunteers. In addition, this study ascertained the clinical utility of each regimen by using the clinician's subjective measure of whether dilation was adequate for his or her examination. We found no difference in the change in resting and constricted pupil size after dilation and there was no difference in the ability of physicians to perform a dilated pupil examination with either regimen. We analyzed several demographic factors that could play a role in the dilatory effect of the drugs. Patient's age, gender, race, or eye color did not have a significant effect on clinical success, signaling the consistency of both drugs to dilate the pupil for diagnostic examination.

While changes in predilation to postdilation pupil size measurements were not significant, we were surprised to find that the patients receiving T+PP achieved greater pupillary dilation compared to PE+T+PP. Similarly there was a statistically significant difference in the proportion of OD eyes that achieved >7 mm dilation between the groups, favoring T+PP. A possible explanation for the larger postdilation pupil size in patients receiving T+PP compared to PE+T+PP includes the order and timing of drop administration. Park et al. reported that 1% tropicamide, with its parasympathetic antagonistic mechanism of action, was more effective at inducing pupillary dilation than 2.5% phenylephrine. In addition, the authors noted that when given independently, the combination of 1% tropicamide and 2.5% phenylephrine was more effective than multiple drops of a single-drug regimen.^22^

In our study, there was only a 15-s duration in between eye drops, which was consistent with the standard clinic protocol. This shortened duration could explain the complex interaction between the eye drops that may have also led to the unexpected finding of T+PP having greater mydriatic efficacy compared to PE+T+PP in the OD eye, but not OS. There may be a “washout” effect from administering multiple drops in the triple regimen group, which could decrease the mydriatic power of each agent.^23^

Our priority was to evaluate efficacy under typical clinical conditions to determine the utility of the third agent in a clinical setting. This includes the use of proparacaine which has been shown to enhance the rate and magnitude of pupillary dilation if instilled before mydriatic agents.^24,25^ The goal of the study was to assess whether the elimination of the expensive phenylephrine drop would negatively impact the ability to conduct a diagnostic examination in standard clinical practice.

We intentionally did not attempt to determine the optimum time between drop administration or order of drops because altering drop administration times or order of drops beyond what is typical in a clinical setting would have created a barrier to clinical acceptance and undermined the study. The results therefore reflect what would be expected in the clinic setting if phenylephrine were eliminated. Interestingly, we find a negligible advantage to the two-drop regimen, suggesting that the typical clinical practice with three drops may not be ideal. Future studies should be conducted to determine whether the effect of duration between drops or order of administration affects pupillary dilation.

A limitation to this study includes the less than anticipated target sample size that was determined using absolute changes in the pupil size. A power analysis based on the magnitude of the difference in pupil size with the two regimens in the study by Liu et al.^4^ suggested a sample size of around 90 patients would be required to test a 20% difference in the magnitude of dilation between the groups, but enrollment fell slightly short of this goal. However, we contend that the efficacy of dilation in both groups suggested that differences between the regimens may actually favor T+PP, but the difference in magnitude does not result in a clinically significant difference in the ability to conduct a diagnostic examination through the dilated pupil.

The main outcome of our study was the clinical efficacy demonstrated by the ability to conduct an examination of the retina by the examining physician without use of additional dilating drops. Based on our results of success rate of 94% for PE+T+PP and 90% for T+PP, a power analysis showed we must enroll 1,442 patients to demonstrate that this is a statistically significant difference in the clinical efficacy of the treatment groups. As a result, we conclude that small differences in the size of the pupils between the regimens do not impact clinical care. This finding is consistent with the hypothesis suggested in the pilot study by Liu et al.^4^ that there is a clinically insignificant benefit to adding phenylephrine to the dilating regimen.

Future studies to address additional factors in the difference between regimens could assess intraindividual comparison of the cocktails in each eye (either PP + T OD and PE+T+PP OS or vice versa). This strategy might strengthen the results of our study that shows T+PP has a greater absolute dilating effect compared to PE+T+PP.

Future studies could also test the validity of the “washout” effect by comparing those who receive the three drops separately with those receiving a cocktail that contains both medications in one vial. Another potential confounder in our study is the effect of iris pigmentation on dilating response. While there were no significant differences in iris pigmentation between the groups in our study and iris color was not a predictive factor for successful clinical examination, individual differences in response to mydriatics could be masked by regression to the mean. The literature suggests that dark eyes take longer to dilate compared to those with lighter pigment—especially in children.^26^

The results of this study and previous studies provide support for single mydriatics agent use with T+PP in comparison to the combination of PE+T+PP. In the setting of rising costs for phenylephrine eye drops, it will be financially prudent to consider using tropicamide as a single agent for the dilated eye examination in clinic, while reserving phenylephrine for resistant cases or pupillary dilation in the OR. As the incidence and prevalence rates for severe eye pathologies continue to increase, it is imperative that physicians look to least expensive alternatives that provide the same efficacy.
